# Three Decades of Managing Pediatric Obstructive Sleep Apnea Syndrome: What’s Old, What’s New

**DOI:** 10.3390/children12070919

**Published:** 2025-07-11

**Authors:** Beatrice Panetti, Claudia Federico, Giuseppe Francesco Sferrazza Papa, Paola Di Filippo, Armando Di Ludovico, Sabrina Di Pillo, Francesco Chiarelli, Alessandra Scaparrotta, Marina Attanasi

**Affiliations:** 1 Pediatric Allergy and Pulmonology Unit, Department of Pediatrics, University of Chieti-Pescara, 66100 Chieti, Italy; beatrice.panetti@studenti.unich.it (B.P.); claudia.federico@studenti.unich.it (C.F.); difilippopaola@libero.it (P.D.F.); armando.diludovico@studenti.unich.it (A.D.L.); sabrina.dipillo@asl2abruzzo.it (S.D.P.); chiarelli@unich.it (F.C.); 2 Department of Neurorehabilitation Sciences, Casa di Cura Igea, 20144 Milan, Italy; g.sferrazza@casadicuraigea.it; 3 Unit of Pediatrics, Local Health Authority (ASL), 65126 Pescara, Italy; a.scaparrotta@asl.pe.it

**Keywords:** pediatric obstructive sleep apnea syndrome, adenotonsillectomy, continuous positive airway pressure, myofunctional therapy, orthodontic interventions

## Abstract

Obstructive sleep apnea syndrome (OSAS) in children and adolescents is a prevalent and multifactorial disorder associated with significant short- and long-term health consequences. While adenotonsillectomy (AT) remains the first-line treatment, a substantial proportion of patients—especially those with obesity, craniofacial anomalies, or comorbid conditions—exhibit persistent or recurrent symptoms, underscoring the need for individualized and multimodal approaches. This review provides an updated and comprehensive overview of current and emerging treatments for pediatric OSAS, with a focus on both surgical and non-surgical options, including pharmacological, orthodontic, and myofunctional therapies. A narrative synthesis of recent literature was conducted, including systematic reviews, randomized controlled trials, and large cohort studies published in the last 10 years. The review emphasizes evidence-based indications, mechanisms of action, efficacy outcomes, safety profiles, and limitations of each therapeutic modality. Adjunctive and alternative treatments such as rapid maxillary expansion, mandibular advancement devices, myofunctional therapy, intranasal corticosteroids, leukotriene receptor antagonists, and hypoglossal nerve stimulation show promising results in selected patient populations. Personalized treatment plans based on anatomical, functional, and developmental characteristics are essential to optimize outcomes. Combination therapies appear particularly effective in children with residual disease after AT or with specific phenotypes such as Down syndrome or maxillary constriction. Pediatric OSAS requires a tailored, multidisciplinary approach that evolves with the child’s growth and clinical profile. Understanding the full spectrum of available therapies allows clinicians to move beyond a one-size-fits-all model, offering more precise and durable treatment pathways. Emerging strategies may further redefine the therapeutic landscape in the coming years.

## 1. Introduction

Obstructive sleep apnea syndrome (OSAS) is characterized by recurrent episodes of partial or complete upper airway obstruction during sleep, leading to intermittent hypoxemia, reduced ventilation, and fragmented sleep architecture. In severe cases, it may contribute to obstructive hypoventilation, particularly in high-risk pediatric populations [1,2,3].

Its prevalence in the pediatric population ranges from 1% to 4%, with an increasing incidence linked to the rising trend of childhood obesity [2,4]. The consequences of untreated OSAS in children are significant and include impaired sleep quality, neurocognitive and behavioral disorders, poor academic performance, delayed neurodevelopment, increased cardiovascular risk, and excessive daytime sleepiness—all of which contribute to a higher burden on healthcare systems [2,3,5,6,7,8,9].

Multiple factors contribute to the development and severity of OSAS, such as obesity, craniofacial abnormalities, and systemic conditions such as genetic syndromes (e.g., Down syndrome, Prader–Willi syndrome) and neurologic/neuromuscular disorders [10,11].

While obstructive and central sleep apnea are distinct entities, mixed sleep-disordered breathing is increasingly recognized, even in otherwise healthy children with OSAS. Central apneas in this context are likely related to arousals from obstructive events and may reflect ventilatory control instability. Notably, adenotonsillectomy (AT) can lead to normalization of both central and mixed apnea indices in a large proportion of affected children, supporting the hypothesis of secondary central dysregulation driven by upper airway obstruction [12,13].

In the pediatric age group, OSAS is frequently associated with adenotonsillar hypertrophy due to a mismatch in growth between lymphoid tissue and the craniofacial skeleton, especially between 5 and 8 years of age [14,15]. AT is commonly considered the first-line treatment, given the tendency for upper airway collapse during sleep due to physiological muscle relaxation [3,16,17,18].

There are multiple treatments for OSAS, and choosing the most appropriate therapeutic approach can often be challenging. When selecting a treatment, the clinician must consider numerous factors to identify the therapy with the greatest efficacy for the patient’s specific underlying condition.

Treatment strategies must be individualized, taking into account the severity of disease, comorbidities, and patient and family preferences. Multidisciplinary evaluation involving pediatric otolaryngology and sleep medicine specialists is essential [2].

In addition, the involvement of other specialists, such as maxillofacial surgeons or orthodontists, may be particularly beneficial for specific subsets of patients.

Hence, this review aims to provide an updated overview of current treatment modalities for pediatric OSAS, offering clinicians practical guidance for tailoring therapy to the unique characteristics of each child.

To our knowledge, this is the first comprehensive review to cover over three decades of therapeutic evolution in pediatric OSAS, integrating surgical, pharmacological, orthodontic, and emerging interventions within a unified clinical and historical framework.

## 2. Materials and Methods

A comprehensive literature search was conducted using PubMed/MEDLINE and Google Scholar to identify relevant studies on pediatric obstructive sleep apnea. To ensure broad international coverage, both American and British spelling variants were included (i.e., “pediatric” and “paediatric”). The primary search terms were: “pediatric obstructive sleep apnea” OR “paediatric obstructive sleep apnea” OR “pediatric sleep-disordered breathing” OR “paediatric sleep-disordered breathing” OR “OSAS” AND “children” OR “adolescents” OR “pediatric” OR “paediatric”.

Additional keyword combinations were used for each specific therapeutic area. A complete list of search terms is provided in Table A1 of Appendix A.

The search, finalized in February 2025, covered a period of approximately 30 years. Included studies were systematic and narrative reviews, longitudinal cohort studies (prospective and retrospective), and randomized controlled trials involving pediatric populations (ages 0–18 years). Only articles published in English were considered. References of selected articles were also screened for additional relevant studies.

This review provides a qualitative synthesis of the literature and did not involve pooled data analysis. The studies included addressed pediatric patients (0–18 years), with or without comorbidities such as craniofacial anomalies, genetic syndromes, or neurologic disorders. Thematic areas of interest included surgical approaches, pharmacological therapy, orthodontic interventions, and non-invasive ventilatory support. Eligible study types encompassed randomized controlled trials, observational studies, and systematic or narrative reviews. No formal statistical analysis was conducted. Literature selection and screening were carried out independently by the authors. As this work is based exclusively on previously published studies, ethical approval was not required.

## 3. Surgical Approaches

### 3.1. Adenotonsillectomy

Adenotonsillectomy (AT) is considered the first-line surgical treatment for pediatric OSAS in children with adenotonsillar hypertrophy. Over the past four decades, its primary indication has evolved from recurrent infections to sleep-disordered breathing, reflecting a growing body of clinical evidence supporting its efficacy [19]. Current systematic reviews, randomized controlled trials (RCTs), and clinical guidelines consistently support AT as the most effective intervention. It improves upper airway patency, sleep architecture, and overall quality of life in affected children. The Clinical Practice Guideline from the American Academy of Otolaryngology–Head and Neck Surgery Foundation affirms that tonsillectomy may be performed with or without adenoidectomy; however, in the context of OSAS, the combined approach remains the standard of care [20].

Venekamp et al. [21] conducted a Cochrane review of multiple RCTs, confirming that AT significantly improves symptoms, quality of life, and polysomnographic outcomes compared to non-surgical management. Among the included studies, the CHAT Trial [22], one of the largest RCTs on this topic, demonstrated polysomnographic normalization in 79% of children undergoing AT, compared to 46% in the watchful waiting group. Additionally, AT significantly reduced daytime sleepiness and improved disease-specific quality of life (OSA-18) [23].

A retrospective analysis by Locci et al. [24] further confirmed AT’s effectiveness, reporting a median reduction in the apnea–hypopnea index (AHI) from 13.4/h to 2.4/h, improved SpO_2_ nadir (from 89% to 94%), and better OSA-18 scores. Similarly, a meta-analysis by Tsikopoulos et al. [25], encompassing 1225 children, found that AT led to significantly greater improvements in AHI and quality of life compared to conservative management.

In recent years, additional high-quality RCTs have further refined our understanding of AT in children with mild OSAS and habitual snoring. The POSTA study (Preschool OSA Tonsillectomy and Adenoidectomy) [26], conducted in preschool-aged children, demonstrated significant improvements in behavioral outcomes and sleep-related quality of life following early surgical intervention compared to watchful waiting. The PATS trial [27], conducted in school-aged children with mild sleep-disordered breathing but no polysomnographic OSAS, found no cognitive benefits, yet reported improvements in symptoms, quality of life, and prevention of OSA progression.

Recently, less invasive surgical alternatives—such as adenotonsillotomy, which entails partial removal of tonsillar tissue—have been evaluated in randomized trials, particularly in preschool-aged children. Two RCTs assessed its long-term effectiveness compared to AT. Borgström et al. [28] showed that adenotonsillotomy was non-inferior in reducing AHI and improving symptoms at 1-year follow-up, although a small subset required reoperation. Sjölander et al. [29] confirmed these results at 5 years, reporting sustained improvements in both groups, but a higher reoperation rate in the adenotonsillotomy group.

Several studies emphasize the importance of including adenoidectomy when treating pediatric OSAS. Children with residual symptoms after tonsillectomy often exhibit persistent adenoid hypertrophy, supporting the necessity of the combined procedure [30,31]. High-risk populations—such as children with Down syndrome—face a greater likelihood of persistent OSAS if adenoidectomy is omitted. Sudarsan et al. demonstrated that AT is particularly beneficial in this group [32].

Despite its benefits, residual OSAS persists in approximately 18–20% of children after AT, especially among those with obesity, craniofacial anomalies, or severe baseline AHI. Obese children may experience continued airway collapse despite surgery [33,34], and those with craniofacial conditions such as midface hypoplasia or trisomy 21 often require additional interventions [35]. A higher preoperative AHI is also predictive of persistent disease [20].

In light of these findings, a risk-stratified postoperative approach is essential. Current recommendations advise repeat polysomnography within 3–6 months in high-risk patients and the initiation of adjunctive treatments—such as CPAP or BiPAP—when residual obstruction is identified [36].

### 3.2. Tonsillectomy

Tonsillectomy remains one of the most frequently performed surgical procedures globally. A variety of techniques have been developed, including classic blunt dissection, guillotine excision, electrocautery, cryosurgery, coblation, ultrasonic and laser-assisted excision, monopolar and bipolar dissection, thermal welding, and ligasure tonsillectomy. These techniques are generally classified into two main categories: extracapsular tonsillectomy (total removal of tonsillar tissue) and intracapsular tonsillectomy (partial/subtotal removal, also known as tonsillotomy). The choice of surgical approach is influenced by factors such as the degree of airway obstruction, the potential for tonsillar regrowth, and the risk of perioperative complications [37]. While extracapsular tonsillectomy remains the conventional standard, particularly in moderate to severe pediatric OSAS, intracapsular tonsillotomy has gained increasing attention due to its favorable short-term outcomes, including reduced postoperative pain, decreased hemorrhagic risk, and more rapid convalescence [38]. However, this technique preserves residual lymphoid tissue, which can regrow in approximately 10–25% of cases, occasionally necessitating revision surgery [39].

In a large retrospective study involving 3127 children with OSAS, Mesolella et al. [40] demonstrated that both extracapsular and intracapsular techniques were effective in alleviating symptoms and improving disease-specific quality of life, which in particular was associated with reduced postoperative morbidity, including lower pain scores and decreased bleeding risk, without evidence of significant regrowth at one-year follow-up. Nevertheless, longer-term outcomes remain a concern. A 2024 meta-analysis encompassing 32 studies and over 9000 pediatric patients confirmed the short-term advantages of tonsillotomy but also identified an increased risk of long-term recurrence and reoperation [41]. The most extensive longitudinal assessment to date, a 12-year prospective study by Virkkunen et al. [42], compared the long-term outcomes of tonsillotomy and tonsillectomy in 778 children. Both approaches yielded sustained improvements in sleep-related quality of life; however, the reoperation rate was significantly higher in the tonsillotomy group (6.9% vs. 1.0%). Furthermore, patients who underwent tonsillotomy reported a higher incidence of airway-related symptoms over time, reinforcing the superior long-term efficacy of complete tonsillar excision.

A prospective five-year study assessing partial intracapsular tonsillectomy using bipolar forceps in combination with adenoidectomy reported a low rate of lymphoid regrowth (2%) and significant improvements in AHI, oxygen saturation, and quality of life metrics [43]. These findings suggest that, when executed with precision, tonsil-preserving techniques may still offer durable outcomes in selected patients.

In summary, although tonsillotomy provides clear short-term advantages—particularly in younger children and those with milder disease—its long-term durability appears inferior to that of extracapsular tonsillectomy. For patients with severe OSAS, markedly enlarged tonsils, obesity, or craniofacial anomalies, complete tonsillar excision remains the more reliable approach for sustained symptom control. The long-term data underscore the necessity of systematic follow-up in children undergoing intracapsular procedures. As surgical strategies evolve, emerging techniques such as subcapsular tonsillectomy may offer an intermediate solution, balancing postoperative morbidity with reduced risk of recurrence. Future randomized controlled trials with extended follow-up are warranted to delineate the comparative efficacy of these techniques and to inform guideline development. Personalized surgical planning, based on individual anatomy, disease severity, and comorbid conditions, is essential for optimizing outcomes. A comparative summary of the current tonsillectomy and tonsillotomy techniques used in pediatric OSAS is presented in Table 1. While AT remains the gold standard for children with adenotonsillar hypertrophy, several evolving approaches—including coblation tonsillotomy, microdebrider-assisted tonsillotomy, and laser-assisted procedures—have demonstrated promising short-term results in well-selected populations. Additionally, transoral robotic surgery (TORS), though not yet widely adopted, offers improved surgical precision and visualization and may be particularly beneficial in patients with craniofacial syndromes or persistent OSAS following traditional AT. Novel modalities such as cryogenic plasma tonsillectomy and radiofrequency ablation (RFA) are also under investigation, though further validation through robust, long-term studies is necessary. Until such evidence is available, the application of these alternative techniques should be individualized based on patient age, anatomical complexity, comorbid conditions, and disease severity.

### 3.3. Adjunctive and Alternative Surgical Procedures for Residual or Complex OSAS

Although AT is effective in most pediatric cases, a subset of children—particularly those with obesity, craniofacial anomalies, neuromuscular disorders, or multilevel airway obstruction—may require additional interventions. Persistent OSAS often involves anatomical sites beyond the tonsils and adenoids, such as the nasal cavity, tongue base, pharyngeal walls, or larynx [44].

Base of tongue surgery, including lingual tonsillectomy and midline posterior glossectomy, has shown promise in children with tongue base hypertrophy or collapse. In a cohort of 168 children undergoing DISE-guided procedures, Williamson et al. [45] reported significant reductions in the obstructive Apnea–Hypopnea Index (oAHI) and a 75% surgical success rate, with minimal complications.

Supraglottoplasty may be indicated in infants with severe laryngomalacia, especially when associated with feeding difficulties or failure to thrive. Verkest et al. [46] observed improvements in oAHI and sleep efficiency following surgery, though recent data suggest that up to 39% of these children may develop recurrent OSAS during follow-up [47].

Uvulopalatopharyngoplasty (UPPP) and expansion sphincter pharyngoplasty (ESP) are generally reserved for children with persistent OSAS following AT, particularly when palatal or lateral pharyngeal wall collapse is confirmed on dynamic airway evaluation. A comprehensive review by Velu et al. [48] documented subjective improvement in 85.6% of children undergoing UPPP, along with a significant reduction in AHI, although outcome definitions varied and complications were not negligible. Similarly, Ulualp [49] reported favorable outcomes with modified ESP in children with DISE-confirmed lateral pharyngeal wall collapse, with no major complications. Conversely, outcomes in obese children appear less favorable: in a study by Com et al. [50], resolution rates with UPPP alone or combined with AT remained below 20%.

Inferior turbinate reduction, often performed alongside AT, may improve nasal airflow and enhance surgical outcomes in children with turbinate hypertrophy. In a prospective study, Cheng et al. [51] demonstrated that AT combined with microdebrider-assisted inferior turbinoplasty yielded greater improvements in polysomnographic and quality-of-life parameters compared to AT alone. These findings were supported by a recent meta-analysis confirming the efficacy of turbinate surgery in enhancing nasal patency [52].

Tracheostomy, although rarely required, remains a definitive intervention in cases of severe, refractory OSAS, particularly in children with syndromic craniofacial anomalies or neuromuscular impairment. In a multicenter study, Rizzi et al. [53] reported 0% tracheostomy-specific mortality and confirmed the procedure’s role in stabilizing the airway. Similarly, Fuller et al. [54] emphasized the importance of comprehensive care, including caregiver education and structured decannulation protocols.

In conclusion, while adjunctive surgical interventions can be valuable in selected cases of residual or complex OSAS, their use should be based on comprehensive airway assessment and individualized risk stratification. Integration of these procedures into a multidisciplinary treatment plan is essential to optimize outcomes in children with treatment-refractory sleep-disordered breathing.

**Figure 1:**
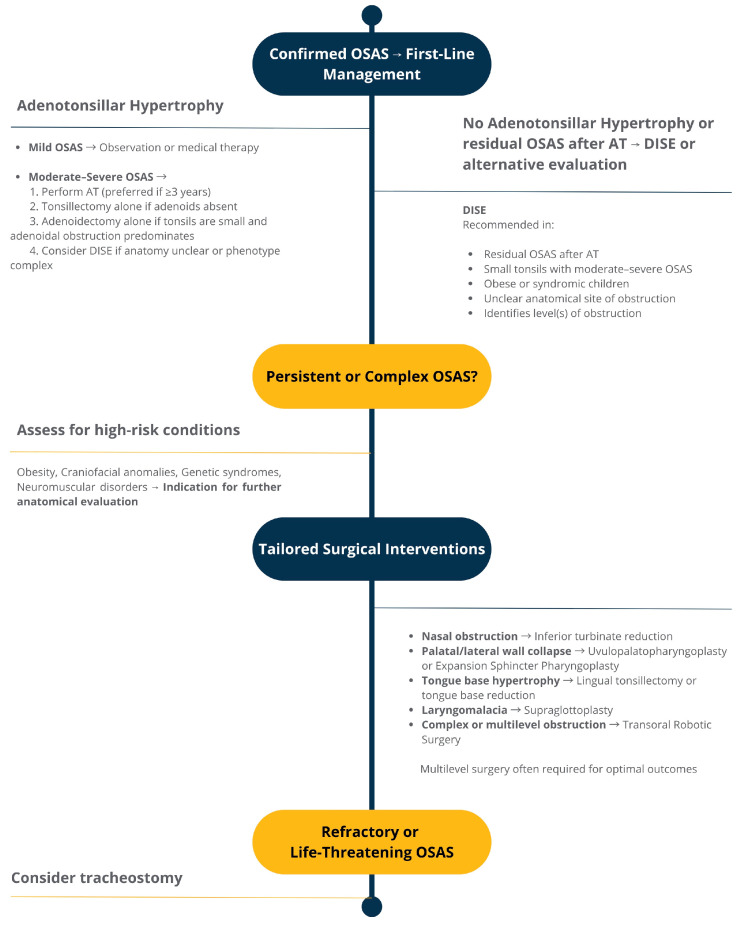
Stepwise Surgical Approach to Pediatric Obstructive Sleep Apnea.

A comprehensive visual summary of surgical decision-making pathways is provided in Figure 1.

This algorithm reflects current expert recommendations and clinical evidence supporting individualized, anatomy-based surgical management. It incorporates the AAO-HNSF (American Academy of Otolaryngology–Head and Neck Surgery Foundation) Expert Consensus Statements on pediatric drug-induced sleep endoscopy (DISE) [55] and post-adenotonsillectomy OSAS [56], which emphasize the role of DISE in persistent or complex cases to guide site-specific interventions.

## 4. Non-Surgical Therapies for Pediatric Obstructive Sleep Apnea

### 4.1. Pharmacological Management

Pharmacological therapy has gained relevance as a non-invasive approach for children with mild OSAS, residual symptoms after surgery, or as a bridging strategy while awaiting specialist evaluation [57]. Among the most studied agents are intranasal corticosteroids (INS) and leukotriene receptor antagonists (LTRA), primarily montelukast [58,59].

#### 4.1.1. Intranasal Corticosteroids (INS)

INS reduce upper airway inflammation by downregulating pro-inflammatory cytokines, which may decrease adenotonsillar hypertrophy and improve airway patency [60]. Clinical trials have demonstrated varying results regarding their efficacy.

A large RCT by Baker et al. [61] evaluated the efficacy of mometasone furoate versus saline nasal spray in 276 children with sleep-disordered breathing. The study demonstrated modest but significant improvements in parent-reported symptoms and sleep quality with INS, although changes in objective sleep study parameters were limited.

Brouillette et al. [62] reported that a 6-week fluticasone course improved AHI without reducing lymphoid tissue size. Similar outcomes were observed with mometasone furoate [63], whereas budesonide led to both AHI reduction and adenoid shrinkage [64].

However, a 2020 Cochrane meta-analysis found that no consistent effect on AHI, oxygen desaturation, or symptom scores was demonstrated [57]. A more recent 12-month RCT confirmed these limitations, showing AHI improvement but no significant changes in neurobehavioral function or quality of life [65]. These findings suggest that INS may offer temporary benefits, but their long-term efficacy as monotherapy is limited.

#### 4.1.2. Leukotriene Receptor Antagonists (Montelukast)

Montelukast acts by inhibiting cysteinyl-leukotriene receptors overexpressed in adenotonsillar tissues, thereby reducing inflammation and tissue hypertrophy [66]. Randomized trials have demonstrated AHI reduction and improved airway patency in children with mild-to-moderate OSAS [67].

The Cochrane review found that montelukast significantly decreased AHI (mean difference −3.41 events/h; moderate-certainty evidence) [57]. Additional studies confirmed improvements in Oxygen Desaturation Index (ODI) and adenoid size [68].

Combining montelukast with INS appears more effective than either alone. A meta-analysis by Liming et al. [69] reported a 70% AHI reduction with combination therapy, compared to 55% with montelukast alone. A 2024 network meta-analysis identified montelukast plus mometasone as the most effective pharmacologic regimen [70].

Despite these benefits, montelukast use must be weighed against potential neuropsychiatric adverse events. In 2020, the FDA issued a black box warning for montelukast, citing risks such as mood changes, aggression, and suicidal ideation [71]. A recent systematic review confirmed increased risk, particularly with prolonged use [72]. Thus, short-term use (≤3 months) may be preferable, especially when combined with INS.

#### 4.1.3. Systemic Corticosteroids and Preoperative Medical Therapy

Systemic corticosteroids have been explored as bridging therapy for children with severe OSAS awaiting surgery. Evangelisti et al. [73] demonstrated that a 7-day course of oral betamethasone combined with INS significantly improved SaO_2_, ODI, and clinical symptoms compared to INS alone. However, due to potential adverse effects (e.g., growth suppression, metabolic changes), systemic corticosteroids should be reserved for select, short-term use.

To conclude, pharmacological therapy represents a promising adjunct or alternative for managing mild-to-moderate pediatric OSAS, particularly in non-surgical candidates or children with residual disease. It has also been assessed preoperatively to potentially reduce surgical necessity. In a cohort of 205 children, medical treatment (INS, montelukast, antiallergics, and antibiotics) improved AHI in mild-to-moderate OSAS but had no impact on the surgical need in severe cases [74]. Notably, the use of antibiotics was adjunctive for treating comorbid infections, not OSAS per se [75]. Montelukast and intranasal corticosteroids, especially in combination, show the greatest efficacy in improving symptoms and selected objective parameters. Nonetheless, limitations regarding long-term outcomes and safety—particularly for montelukast—underscore the need for cautious, individualized use. Further high-quality RCTs are essential to clarify optimal treatment duration, safety profiles, and role in routine practice.

### 4.2. Respiratory Support: CPAP and Beyond

#### 4.2.1. Non-Invasive Ventilation

Adenotonsillar hypertrophy is the most common cause of upper airway obstruction in children, and AT remains the first-line treatment for OSAS [23]. However, surgery is not always feasible or curative. Risk factors for surgical failure or complications include age under 3 years, severe OSAS, obesity, failure to thrive, and the presence of comorbidities such as craniofacial anomalies, genetic syndromes, and neuromuscular diseases [1]. Children who are not candidates for surgery, who show residual OSAS after AT, or whose anatomical features do not support surgical correction often require alternative therapeutic approaches. In these cases, positive airway pressure (PAP) therapy is the mainstay of non-surgical management [76]. PAP can be delivered as continuous (CPAP), bilevel (BPAP), or auto-adjusting (APAP) pressure. Among these, CPAP is the most commonly used and well-established modality in pediatric OSAS. It works by maintaining a constant pressure throughout the respiratory cycle, preventing upper airway collapse [77]. CPAP is typically administered via nasal or oronasal interfaces, with improved tolerability owing to advances in mask design and humidification systems, which help reduce nasal dryness, air leaks, and pressure intolerance [78].

Initiation of CPAP usually involves manual titration during an in-laboratory polysomnography (PSG), which remains the gold standard for determining the optimal therapeutic pressure and assessing treatment efficacy [79]. However, due to availability, outpatient titration of PAP is widely used. Adjustments over time are often required due to growth, weight changes, or evolving upper airway anatomy.

According to the 2012 American Academy of Pediatrics (AAP) guidelines, CPAP is recommended for children with persistent OSAS after AT or when surgery is not indicated [1]. Residual OSAS is relatively common, affecting 13–29% of low-risk children and up to 73% of obese patients, depending on the AHI threshold used. CPAP is also appropriate in the absence of hypertrophic adenotonsillar tissue or when parents decline surgical intervention. Furthermore, CPAP is indicated in children with significant nocturnal hypoxemia, frequent arousals, or sustained hypercapnia on PSG, especially when the AHI is ≥5 events/h [1]. The goals of CPAP therapy are to maintain airway patency, reduce sleep fragmentation and oxygen desaturation, and improve daytime functioning. Several studies have confirmed improvements in daytime sleepiness, behavior, attention, and overall quality of life, even with suboptimal nightly usage [80]. Despite its benefits, adherence to PAP therapy remains a major limitation in pediatric populations. A systematic review by Watach et al. [81] showed average adherence rates of 56.9%, with nightly use ranging from 4.0 to 5.2 h. Common barriers include mask discomfort, air leaks, nasal congestion, behavioral resistance, and caregiver attitudes. Children with developmental disabilities or medical fragility often exhibit higher adherence, while adolescents tend to struggle more [82,83]. The COVID-19 pandemic further influenced adherence patterns, with some children increasing nightly use, while others discontinued therapy [84,85]. Compared to adults, sustaining long-term PAP therapy in children presents additional challenges. Pediatric patients depend heavily on caregivers, making parental engagement (“buy-in”) essential for adherence. Furthermore, successful initiation and maintenance of therapy require experienced healthcare professionals familiar with pediatric behavioral needs and family-centered care—support structures that are generally not needed for adult patients [86].

Importantly, in children, regular follow-up is essential to assess evolving pressure requirements, ensure optimal mask fit as the child grows, and evaluate for potential spontaneous resolution of OSAS with age. These dynamic changes underscore the importance of individualized, ongoing care plans when prescribing PAP therapy in the pediatric population.

Auto-adjusting PAP (APAP) automatically modifies pressure levels based on airflow dynamics. While its use is widespread in adults, pediatric studies have shown variable results. APAP can be less tolerated, with adherence issues and inconsistent pressure delivery [87,88].

Auto-adjusting positive airway pressure (APAP) automatically modifies pressure levels based on airflow dynamics. While widely used in adults, pediatric studies have shown variable results. In children, APAP may be less well tolerated, with issues related to adherence and inconsistent pressure delivery [87,88]. Moreover, its use is limited by manufacturer guidelines, which often specify minimum age or weight thresholds—thereby restricting its applicability, particularly in younger or underweight patients. BPAP is recommended in cases of OSAS associated with hypoventilation, particularly in children with neuromuscular disorders or obesity hypoventilation syndrome. Unlike CPAP, BPAP provides separate inspiratory and expiratory pressures, improving gas exchange and reducing respiratory effort [89,90]. BPAP modes include spontaneous (BPAP-S), spontaneous-timed (BPAP-ST), auto-BPAP, and volume-assured pressure support (VAPS), tailored to specific clinical scenarios. Initiating BPAP therapy in children can be particularly challenging. Achieving effective patient–ventilator synchrony often requires specialized expertise, both in device programming and in behavioral support, to ensure comfort and adherence. Pediatric patients may have difficulty tolerating the complex pressure settings, further emphasizing the need for experienced multidisciplinary teams.

#### 4.2.2. High-Flow Nasal Cannula

High-flow nasal cannula (HFNC) has emerged as a potential alternative to PAP in infants and CPAP-intolerant children. By delivering heated, humidified air at high flow rates, HFNC generates a mild positive pressure, improves oxygenation, and enhances comfort. Early studies showed moderate reductions in AHI and increased oxygen saturation [91,92]. A randomized trial demonstrated that CPAP remained more effective than HFNC in reducing AHI, though HFNC was better tolerated [93]. The 2024 ERS update supports the use of HFNC as a bridge therapy in selected infants with persistent OSAS or CPAP intolerance [94].

Recent ERS statements from 2016 and 2022 emphasize that CPAP, BPAP, and HFNC should be included within a personalized, multidisciplinary framework, especially in children with complex OSAS phenotypes [95,96]. In particular, NIV is essential for patients with neuromuscular disorders, craniofacial anomalies, or progressive respiratory load imbalance. Regular PSG-based reassessment is recommended, typically on an annual basis or more frequently in high-risk patients.

### 4.3. Orthodontic and Myofunctional Interventions

While traditional treatments such as AT and CPAP therapy remain widely used, orthodontic and myofunctional therapies are increasingly considered potential adjuncts or alternatives, particularly in cases of mild to moderate pediatric OSAS [97].

However, current evidence supporting these interventions remains limited. Most published studies are based on small sample sizes, with significant heterogeneity in design and outcomes.

Recent literature emphasizes the potential of orthodontic interventions such as rapid maxillary expansion (RME) and mandibular advancement appliances (MAA) in modifying airway morphology and reducing obstructive events through skeletal remodeling that improves oropharyngeal patency [98]. RME is primarily indicated for maxillary transverse deficiency, nasal obstruction, or high-arched palates [99], while MAA are suitable for patients with mandibular retrognathia or oropharyngeal crowding [100].

RME expands the midpalatal suture and increases nasal cavity volume, enhancing airflow and improving respiratory function [101]. Several studies have demonstrated significant improvements in airway patency and AHI following RME [102]. Additionally, RME has been associated with reductions in adenotonsillar hypertrophy, potentially by restoring nasal breathing and reducing chronic oropharyngeal inflammation [103].

Given that up to 25% of children may experience residual OSAS post-AT [104], RME serves as a particularly relevant adjunctive treatment, especially in patients with craniofacial anomalies.

MAA act by repositioning the mandible and tongue base anteriorly, thus enlarging the hypopharyngeal space [105]. These appliances have shown efficacy in reducing AHI and improving oxygen saturation, particularly in children intolerant to CPAP therapy [106]. MAA have also been linked to improvements in cognitive function, behavior, and quality of life [107].

Despite their benefits, MAA are limited by adherence challenges and potential side effects such as temporomandibular joint discomfort. Custom-fitted, adjustable devices are being developed to improve comfort and compliance [108], though long-term data on treatment stability remain scarce [109]. In contrast, RME appears to offer more stable long-term benefits, with evidence of sustained airway improvement up to 36 months post-treatment [110].

Nevertheless, the evidence base for both mandibular advancement and maxillary expansion remains limited and should be interpreted with caution. Ongoing research is promising, and future large-scale, well-designed RCTs will provide evidence and better define their role in clinical practice [100].

Combining MAA with RME or with AT may provide enhanced outcomes. A network meta-analysis showed that combined treatments (e.g., RME + MAA or RME + AT) led to greater AHI reduction than MAA alone [111]. Myofunctional therapy (MT), involving oropharyngeal exercises to strengthen airway musculature and promote nasal breathing, has been shown to reduce snoring and AHI [112]. MT enhances tongue motor control and endurance, which are essential for maintaining airway patency [113]. Integration with orthodontic treatments may further improve outcomes, especially when initiated early in craniofacial development [114,115].

Passive myofunctional therapy (PMFT), using oral appliances to guide tongue posture, has also shown efficacy in reducing AHI and improving oxygen saturation [116]. Some studies suggest that MT may also prevent residual OSAS post-AT and enhance CPAP adherence [117], though further trials are needed to standardize protocols and assess long-term outcomes. Similar limitations apply to myofunctional therapies. As noted by Saba et al. [115], more studies are required to evaluate compliance and the long-term effects of OMT on OSA outcomes.

Importantly, the use of RME and MAA in children should always involve specialized orthodontic or craniofacial expertise to ensure proper patient selection, treatment planning, and follow-up.

In conclusion, orthodontic and myofunctional therapies represent promising components of a multimodal treatment strategy for pediatric OSAS. In selected patients, especially those with craniofacial abnormalities or CPAP intolerance, these interventions may offer sustainable improvements. Notably, orthodontic treatments may benefit children with neurological impairments as well. A case study reported substantial AHI and oxygenation improvements in a child with cerebral palsy following at-home orthodontic therapy [118]. While encouraging, further long-term and controlled studies are needed to confirm the durability and generalizability of these outcomes.

## 5. Innovative and Investigational Treatments for Pediatric Obstructive Sleep Apnea

Hypoglossal nerve stimulation (HNS) represents an innovative neuromodulation approach for pediatric OSAS, particularly in children with Down syndrome (DS), a population characterized by an elevated prevalence of OSAS (55–97%), high residual disease rates post-AT, and limited CPAP adherence [119]. HNS delivers synchronized stimulation to the hypoglossal nerve during inspiration, promoting tongue protrusion and airway patency. The system includes an implantable pulse generator, a respiratory sensing lead, and a stimulation cuff. Unlike CPAP, HNS targets the neuromuscular pathophysiology of OSAS [120].

A 2022 meta-analysis of nine studies involving 106 adolescents with DS found that HNS reduced AHI by a mean of 17.43 events/h and improved quality of life; 65.9% achieved ≥50% AHI reduction, and 73.2% reached an AHI < 10 events/h [121]. A long-term follow-up study reported sustained benefits up to 58 months post-implantation, with AHI worsening upon device deactivation [122].

A recent systematic review added qualitative insights, highlighting improvements in daytime alertness, adaptive behavior, and parental satisfaction [123,124]. Despite these benefits, device-related complications were reported in 56% of children and were primarily minor, but occasionally required reoperation, such as battery replacement [125].

It is important to note that HNS requires specialized surgical and sleep medicine expertise for patient selection, implantation, and titration. Moreover, this technology is not yet widely available in all countries, which may limit access and implementation in routine pediatric care.

While HNS shows promise in DS populations, its applicability to other pediatric cohorts remains to be established. Future research should assess its efficacy in children with obesity or craniofacial syndromes and evaluate the cumulative burden of device maintenance.

Positional therapy is an emerging strategy for children with positional obstructive sleep apnea, characterized by a worsening of obstructive events in the supine position. A recent randomized controlled crossover trial by Xiao et al. [126] investigated the use of positional devices in 66 children and found a significant reduction in AHI when supine sleep was avoided. Improvements were also observed in sleep architecture and parental sleep quality. These findings suggest that positional therapy may represent a valuable, low-risk option for selected pediatric patients, particularly those with mild positional OSAS or those awaiting more definitive treatment.

Emerging neuromodulation targets, such as the ansa cervicalis, are under investigation, though current evidence is limited to adult studies [127].

Novel pharmacologic approaches also show potential. A pilot trial by Combs et al. tested a combination of atomoxetine and oxybutynin in 15 children with DS and moderate OSAS, resulting in a 47–51% reduction in AHI with good adherence and minimal adverse effects [128]. An ongoing trial (NCT05933603) is evaluating this combination over a six-month period, incorporating sleep-related quality of life and cognitive outcomes.

In adults, carbonic anhydrase inhibitors such as sulthiame have demonstrated efficacy in reducing respiratory events by stabilizing ventilatory control [129]. These agents induce mild metabolic acidosis, thereby enhancing respiratory chemosensitivity [130,131]. While pediatric application is still theoretical, sulthiame’s prior use in pediatric neurology suggests feasibility pending dedicated trials.

In summary, neuromodulation, positional therapy, and novel pharmacologic therapies offer promising adjuncts or alternatives in pediatric OSAS, particularly for patients with residual disease or poor adherence to conventional treatments. However, broader, long-term trials across diverse pediatric populations are needed to establish safety, efficacy, and integration into clinical algorithms.

**Figure 2:**
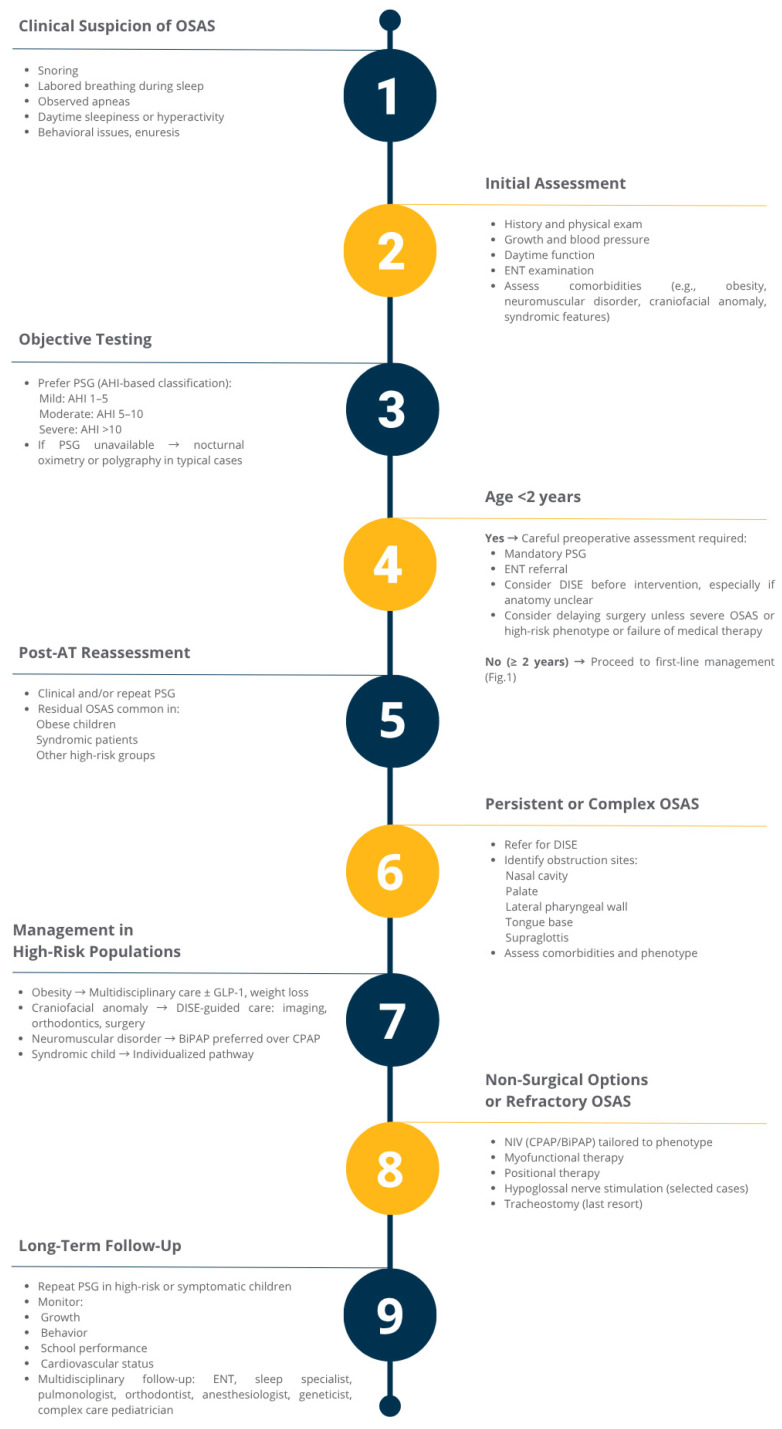
Diagrammatic Clinical Pathway for Pediatric OSAS Management.

To conclude, a multidisciplinary algorithm integrating diagnostic steps and personalized management strategies is proposed by the authors and shown in Figure 2.

## 6. Conclusions

Pediatric obstructive sleep apnea is a multifaceted disorder that requires equally complex and individualized management strategies. From surgical innovations to emerging pharmacologic and orthodontic therapies, the expanding therapeutic arsenal offers promising opportunities—yet no one-size-fits-all approach exists. Each pediatric patient with OSAS should receive a personalized treatment plan tailored to their age, comorbidities, disease severity, and ability to adhere to or tolerate specific therapies.

## Figures and Tables

**Table 1:** Comparative overview of surgical techniques for tonsillectomy and tonsillotomy in the management of pediatric OSAS.

Surgical Technique	Mechanism	Key Advantages	Limitations	Typical Indications
**Cold Steel Dissection**	Dissection of the tonsillar capsule using scalpel and scissors; exposes pharyngeal muscles	Cost-effective, traditional approach	Higher postoperative pain and hemorrhage risk	Standard pediatric OSAS without complicating factors
**Electrocautery** **Tonsillectomy**	Monopolar or bipolar cautery (temperatures up to 400 °C) enabling dissection and hemostasis	Reduced operative time, effective bleeding control	Greater thermal damage to adjacent tissues	Routine tonsillectomy when equipment is available
**Harmonic Scalpel** **Tonsillectomy**	Ultrasonic energy simultaneously cuts and coagulates at lower temperatures	Lower thermal spread, reduced postoperative pain	Costly, less widespread availability	Centers equipped with harmonic technology
**Coblation Tonsillotomy**	Uses a plasma field (40–70 °C) generated by radiofrequency and saline	Minimal tissue trauma, low intraoperative bleeding	Higher cost, possible regrowth in younger patients	Favorable in intracapsular tonsillectomy in children
**Microdebrider-Assisted Intracapsular** **Tonsillotomy**	Rotating blade preserves tonsillar capsule while removing hypertrophic tissue	Less pain, faster recovery, low bleeding	May be suboptimal in massive hypertrophy	Mild/moderate OSAS or patients needing rapid return to activity
**Laser-Assisted Serial Tonsillectomy (LAST)**	CO_2_ or diode laser vaporizes tonsillar tissue gradually	Lower intraoperative bleeding, shorter healing time	Risk of incomplete removal, recurrence	Selected cases, limited pediatric application
**Radiofrequency** **Ablation (RFA)**	Submucosal tissue reduction via low-energy RF waves	Immune tissue sparing, reduced pain	Limited evidence in pediatric OSAS	Younger children with immune considerations
**Cryo-Tonsillectomy**	Cryogenic tissue destruction using liquid nitrogen (−196 °C)	Minimal bleeding	Longer operative time, delayed healing	Rarely employed in modern practice
**Cryogenic Plasma** **Tonsillectomy**	Cold plasma ablation reduces thermal injury and preserves mucosa	Less trauma, good postoperative oxygenation	Specialized equipment, limited data	Promising in selected or recurrent cases
**Transoral Robotic** **Surgery (TORS)**	Robotic-assisted precision excision, typically for lingual or residual tonsillar tissue	Enhanced visualization, high precision in anatomically complex cases	High cost, specialized training, longer operative time	Refractory OSAS after AT, especially in syndromic patients

Abbreviations: AT: adenotonsillectomy; OSAS: obstructive sleep apnea syndrome; AHI: apnea–hypopnea index; RFA: radiofrequency ablation; TORS: transoral robotic surgery; LAST: laser-assisted serial tonsillectomy.

## Data Availability

No new data were created.

## Abbreviations

The following abbreviations are used in this manuscript:

AHI	Apnea–Hypopnea Index
APAP	Auto-adjusting Positive Airway Pressure
AT	Adenotonsillectomy
BPAP	Bilevel Positive Airway Pressure
CPAP	Continuous Positive Airway Pressure
DS	Down Syndrome
ENT	Ear, Nose, and Throat
ESP	Expansion Sphincter Pharyngoplasty
HFNC	High-Flow Nasal Cannula
HNS	Hypoglossal Nerve Stimulation
INS	Intranasal Corticosteroids
LTRA	Leukotriene Receptor Antagonists
MAA	Mandibular Advancement Appliances
MT	Myofunctional Therapy
OSAS	Obstructive Sleep Apnea Syndrome
PAP	Positive Airway Pressure
PMFT	Passive Myofunctional Therapy
PSG	Polysomnography
RCT	Randomized Controlled Trial
RFA	Radiofrequency Ablation
RME	Rapid Maxillary Expansion
ST	Spontaneous-Timed mode
TORS	Transoral Robotic Surgery
UPPP	Uvulopalatopharyngoplasty

## Appendix A

**Table A1:** Search Terms by Therapeutic Area.

Therapeutic Area	Search Terms
Surgery	“surgery” OR “adenotonsillectomy” OR “tonsillectomy” OR “surgical outcomes in pediatric OSAS”
Pharmacological Therapy	“drug therapy pediatric OSAS” OR “intranasal corticosteroids and OSAS” OR “montelukast pediatric OSAS” OR “anti-inflammatory therapy for sleep apnea”
Non-Invasive Ventilation	“pediatric CPAP” OR “paediatric CPAP” OR “CPAP adherence children” OR “CPAP efficacy in pediatric OSAS” OR “long-term CPAP outcomes” OR “non- invasive ventilation pediatric OSAS” OR “BiPAP children OSAS” OR “high-flow nasal cannula pediatric OSAS” OR “HFNC children sleep apnea” OR “high flow therapy pediatric sleep-disordered breathing”
Orthodontic Interventions	“rapid maxillary expansion OSAS” OR “mandibular advancement devices pediatric OSAS” OR “myofunctional therapy sleep-disordered breathing”
Emerging Treatments	“alternative pharmacological treatments for OSAS” OR “novel treatments pediatric OSAS” OR “hypoglossal nerve stimulation children”
